# County-Level Social Vulnerability and Breast, Cervical, and Colorectal Cancer Screening Rates in the US, 2018

**DOI:** 10.1001/jamanetworkopen.2022.33429

**Published:** 2022-09-27

**Authors:** Cici Bauer, Kehe Zhang, Qian Xiao, Jiachen Lu, Young-Rock Hong, Ryan Suk

**Affiliations:** 1 Department of Biostatistics and Data Science, The University of Texas Health Science Center at Houston School of Public Health, Houston; 2 Department of Epidemiology, Human Genetics and Environmental Health, The University of Texas Health Science Center at Houston School of Public Health, Houston; 3 Department of Health Services Research, Management and Policy, College of Public Health and Health Professions, University of Florida, Gainesville; 4 UFHealth Cancer Center, Gainesville, Florida; 5 Department of Management, Policy and Community Health, The University of Texas Health Science Center at Houston School of Public Health, Houston

## Abstract

**Importance:**

Area-level factors have been identified as important social determinants of health (SDoH) that impact many health-related outcomes. Less is known about how the social vulnerability index (SVI), as a scalable composite score, can multidimensionally explain the population-based cancer screening program uptake at a county level.

**Objective:**

To examine the geographic variation of US Preventive Services Task Force (USPSTF)–recommended breast, cervical, and colorectal cancer screening rates and the association between county-level SVI and the 3 screening rates.

**Design, Setting, and Participants:**

This population-based cross-sectional study used county-level information from the Centers for Disease Control and Prevention’s PLACES and SVI data sets from 2018 for 3141 US counties. Analyses were conducted from October 2021 to February 2022.

**Exposures:**

Social vulnerability index score categorized in quintiles.

**Main Outcomes and Measures:**

The main outcome was county-level rates of USPSTF guideline-concordant, up-to-date breast, cervical, and colorectal screenings. Odds ratios were calculated for each cancer screening by SVI quintile as unadjusted (only accounting for eligible population per county) or adjusted for urban-rural status, percentage of uninsured adults, and primary care physician rate per 100 000 residents.

**Results:**

Across 3141 counties, county-level cancer screening rates showed regional disparities ranging from 54.0% to 81.8% for breast cancer screening, from 69.9% to 89.7% for cervical cancer screening, and from 39.8% to 74.4% for colorectal cancer screening. The multivariable regression model showed that a higher SVI was significantly associated with lower odds of cancer screening, with the lowest odds in the highest SVI quintile. When comparing the highest quintile of SVI (SVI-Q5) with the lowest quintile of SVI (SVI-Q1), the unadjusted odds ratio was 0.86 (95% posterior credible interval [CrI], 0.84-0.87) for breast cancer screening, 0.80 (95% CrI, 0.79-0.81) for cervical cancer screening, and 0.72 (95% CrI, 0.71-0.73) for colorectal cancer screening. When fully adjusted, the odds ratio was 0.92 (95% CrI, 0.90-0.93) for breast cancer screening, 0.87 (95% CrI, 0.86-0.88) for cervical cancer screening, and 0.86 (95% CrI, 0.85-0.88) for colorectal cancer screening, showing slightly attenuated associations.

**Conclusions and Relevance:**

In this cross-sectional study, regional disparities were found in cancer screening rates at a county level. Quantifying how SVI associates with each cancer screening rate could provide insight into the design and focus of future interventions targeting cancer prevention disparities.

## Key Points

**Question:**

What is the association between social vulnerability and breast, cervical, or colorectal cancer screening rates at the county level in the US?

**Findings:**

In this cross-sectional study of 3141 counties in the US, the US Preventive Services Task Force guideline-concordant screening rates of each cancer showed regional disparities. US counties with higher social vulnerability—measured as a composite index score—had significantly lower odds of receiving the recommended cancer screenings.

**Meaning:**

These findings suggest that geographically targeted public health interventions could be further informed and improved by a composite measure reflecting the multidimensional nature of area-level social determinants of health.

## Teaser

This cross-sectional study examines the association between county-level social vulnerability and rates of screening for breast, cervical, and colorectal cancer in the US.

## Introduction

In the US, breast cancer, cervical cancer, and colorectal cancer could be prevented using the cancer screening schedules recommended by the US Preventive Services Task Force (USPSTF), with the highest level of recommendation (A or B grade).^1,2,3^ These cancer screening recommendations, focusing on the general population at average risk, have been the driving force for the declining population-level incidence and mortality of these cancer types in the US.^4,5,6^ For optimal prevention outcomes at the population level, adequate screening uptake is crucial; however, the screening rates for these 3 cancers are below target rates.^7,8,9^

Because these screening strategies are universal, target average-risk populations, and are not risk based, assessing the uptake of these prevention measures might help identify underserved populations in general. Therefore, understanding how social determinants of health (SDoH) are associated with population-based cancer screening uptake can provide an opportunity to understand how SDoH contribute to health care utilization in diverse populations. Social determinants of health encompass a hierarchical structure of factors at both individual and contextual (eg, physical and social environment of various geographic scales) levels.^10^ Unlike individual-level determinants, area-level determinants capture the conditions that commonly affect all individuals residing in the same area. Understanding these contextual effects could help identify modifiable risk factors that are amenable to public health policies and population- or area-focused interventions^11^ and is therefore crucial for developing effective screening uptake policies.

Of area-level SDoH measures, the social vulnerability index (SVI), developed by the Centers for Disease Control and Prevention (CDC), quantifies the area-level social vulnerability at the US county and Census tract levels.^12^ It has been used to identify areas most at risk during hazardous events and communities with the least infrastructure, fewest resources, and least access to health care. Recently, SVI has also been shown to be associated with certain health services utilization and health-related outcomes (eg, COVID-19 incidence and mortality, obesity, and surgery utilization).^13,14,15,16,17^ Measures used to construct SVI include socioeconomic status, household composition and disability, minority status and language, housing type, and transportation. These factors, when individually assessed, are associated with cancer screening uptake both at individual and area levels.^18,19,20,21,22,23,24,25,26,27,28,29,30^ However, SDoH is intertwined in nature and cannot be explained through a single factor. Less is known about how SVI, a scalable composite score that reflects the multidimensional nature of SDoH, can be useful to explain the population-based cancer screening program uptake and existing disparities. To address this gap in the literature, we examined the geographic variation of USPSTF-recommended breast, cervical, and colorectal cancer screening rates and their associations with county-level SVI in 3141 US counties for 2018. We also analyzed to what degree other measurements not captured by SVI (eg, rural-urban status, health insurance coverage, and density of primary care physicians) are associated with the screening rates.

## Methods

### Data Sources

We conducted a cross-sectional study using the cancer screening rates extracted from the PLACES data set published by the CDC for 2018. PLACES provides estimations of 29 chronic disease health outcomes and health behaviors at the county, Census tract, place (ie, cities), and ZIP Code Tabulation Areas in the US. These estimates were based on the Behavioral Risk Factor Surveillance System public use data and US Census data and obtained using multilevel regression and poststratification approaches for small area estimation.^31^ County-level social vulnerability (SVI) was obtained from the CDC for 2018.^12^ The SVI was constructed using 15 county-level demographic and socioeconomic variables from the American Community Survey and was provided as an index score. Detailed descriptions of the data sets and the methodology can be found on the CDC’s PLACES and SVI websites.^12,31^ To account for measures not included in the SVI but that may contribute to geographic variation, we also included county-level Rural-Urban Continuum Codes,^32^ percentage of uninsured adults,^33^ and numbers of primary care physicians per 100 000 population,^34^ all at the county level (eFigure 1 in the Supplement). All data were at a county level without individual information and were publicly available; thus, this study was deemed exempt from institutional review board approval by The University of Texas Health Science Center. Analyses were conducted from October 2021 to February 2022. This study followed the Strengthening the Reporting of Observational Studies in Epidemiology (STROBE) reporting guideline for cross-sectional studies.

### Measures

We included 3 outcome variables measuring the county-level, up-to-date cancer screening uptake: breast cancer screening, cervical cancer screening, and colorectal cancer screening. All screening rates were age adjusted to the 2000 US standard population.^35^ Up-to-date breast cancer screening was measured as the age-adjusted prevalence of participants who received mammography within the past 2 years among women aged 50 to 74 years.^35^ Cervical cancer screening rate was measured as the age-adjusted prevalence of women aged 21 to 29 years who reported receiving cytology within the past 3 years and women aged 30 to 65 years who reported receiving cytology alone within the past 3 years or human papillomavirus testing or cotesting within the past 5 years among women aged 21 to 65 years without a hysterectomy.^35^ Colorectal cancer screening rate was measured as age-adjusted prevalence by a fecal occult blood test (within the past year), sigmoidoscopy (within the past 5 years with fecal occult blood test within the past 3 years), or colonoscopy (within the past 10 years) among adults aged 50 to 75 years.^35^ These screening rates were model-based estimates accounting for the complex survey design in the Behavioral Risk Factor Surveillance System.

We considered county-level SVI as the primary exposure variable. All SVI scores are presented by ranks on a scale from 0 to 1, with higher values indicating higher social vulnerability. We categorized the SVI into categories (ie, 0 to <0.2, 0.2 to <0.4, 0.4 to <0.6, 0.6 to <0.8, and 0.8 to 1) closely corresponding to their quintiles, which we referred to as Q1 to Q5 throughout the analysis, with Q1 serving as the reference group (ie, the least socially vulnerable). For other covariates, we dichotomized the original 9 Rural-Urban Continuum Code categories into rural (codes 4-9; nonmetro) and urban (codes 1-3; metro). County-level percentage of uninsured population and number of primary care physicians per 100 000 population were also used, which we scaled to have a mean of 0 and a standard deviation of 1 for all counties. We also included the county-level percentage of the eligible population for the given screening outcome to account for differences in the underlying population size.

### Statistical Analysis

We first calculated summary statistics for each screening rate and other county-level covariates stratified by the SVI categories. We then fit a bayesian mixed-effects beta model to evaluate the association between each screening rate and SVI, which was quantified by the estimated odds ratio (OR) (relative to the reference group Q1). Because all 3 screening rates were reported in percentages bounded between 0 and 1, we chose a beta distribution when modeling the rate *p_i_* for the *i*th county. The mean function *μ_i_* of the beta distribution was then decomposed into a state-level fixed effect, a fixed effect with SVI categories, and a county-level random effect. The purpose of the county-level random effect was to account for any additional unmeasured county-level factor (eg, local policy difference), and it was assumed to have a normal distribution. The priors were chosen as commonly used noninformative priors, and the statistical significance was assessed using 95% posterior credible intervals (95% CrIs). We developed a series of models as follows: when investigating the association between SVI and each cancer screening rate, we only adjusted for the eligible population size in model 1 and additionally adjusted for the county urban-rural status in model 2. Model 3 was the full model and further adjusted for health care access (percentage of uninsured population and primary care physicians per 100 000 population). Additional details for model equations are reported in the eMethods in the Supplement. All bayesian models were implemented in R, version 4.0.4 (R Foundation) and R package INLA.^36,37^

## Results

The county characteristics by SVI groups from Q1 (least vulnerable) to Q5 (most vulnerable) are presented in the Table. Of 3141 counties, 1974 (62.8%) were classified as rural counties, with an increasing proportion from SVI-Q1 (59.9%) to Q5 (74.5%). The median percentage of uninsured population was 10.6% (range, 2.4%-32.2%) and also presented an increasing trend with SVI, with median values of 7.9% (range, 2.5%-22.0%) in SVI-Q1 and 14.9% (range, 3.8%-32.1%) in SVI-Q5. The rate of primary care physicians per 100 000 population showed a reverse association with SVI, with higher rates among the less socially vulnerable groups (Q1 = 61.4 vs Q5 = 43.9 primary care physicians per 100 000 population).

**Table.** County-Level Descriptive Summary by Social Vulnerability Index (SVI) Quintile Categories in 2018

	SVI categories^a^					
	0 to <0.2	0.2 to <0.4	0.4 to <0.6	0.6 to <0.8	0.8 to 1	Overall
No. of counties	629	628	628	628	628	3141
**Breast cancer screening**						
Mean (SD)	72.0 (3.6)	71.3 (3.9)	70.5 (3.9)	70.2 (3.8)	69.8 (4.5)	70.8 (4.0)
Median (range)	72.1 (59.8-81.3)	71.5 (58.6-80.3)	70.4 (59.5-81.5)	70.0 (59.9-81.8)	69.8 (54.0-81.2)	70.8 (54.0-81.8)
**Cervical cancer screening**						
Mean (SD)	85.3 (1.4)	84.5 (1.6)	83.8 (1.6)	83.3 (1.6)	82.5 (2.0)	83.9 (1.9)
Median (range)	85.1 (80.7-89.7)	84.4 (69.9-89.2)	83.7 (73.8-88.3)	83.2 (76.5-88.3)	82.6 (74.3-88.1)	84.0 (69.9-89.7)
**Colorectal cancer screening**						
Mean (SD)	64.8 (4.0)	63.7 (3.9)	62.5 (3.9)	60.9 (3.8)	57.7 (4.6)	61.9 (4.8)
Median (range)	64.8 (53.0-74.4)	63.7 (51.9-73.7)	62.4 (49.7-73.6)	60.9 (45.7-73.1)	58.2 (39.8-69.0)	62.1 (39.8-74.4)
**Urban-rural indicator, No. (%)**						
Rural	377 (59.9)	358 (57.0)	375 (59.7)	395 (62.9)	468 (74.5)	1974 (62.8)
Urban	252 (40.1)	270 (43.0)	253 (40.3)	233 (37.1)	158 (25.2)	1166 (37.1)
Missing	0	0	0	0	2 (0.3)	2 (0.1)
**% Uninsured^b^**						
Mean (SD)	8.4 (3.2)	9.7 (3.9)	11.6 (4.9)	12.8 (4.9)	15.1 (5.1)	11.5 (5.0)
Median (range)	7.9 (2.5-22.0)	8.92 (2.4-29.6)	10.7 (3.1-30.2)	12.3 (4.4-32.2)	14.9 (3.8-32.1)	10.6 (2.4-32.2)
Missing, No. (%)	0	1 (0.2)	0	0	0	1 (0.0)
**Access to primary care^c^**						
Mean (SD)	61.4 (44.0)	60.3 (40.5)	55.8 (33.8)	50.0 (29.7)	43.9 (25.6)	54.3 (36.0)
Median (range)	53.7 (0.0-436)	53.0 (0.0-559)	48.2 (0.0-291)	45.6 (0.0-256)	40.4 (0.0-260)	47.5 (0.0-559)
Missing, No. (%)	40 (6.4)	21 (3.3)	28 (4.5)	22 (3.5)	39 (6.2)	150 (4.8)

^a^ Scores are presented on a scale from 0 to 1, with higher values indicating higher social vulnerability.

^b^ Indicates the proportion of adults aged 18 to 64 years who currently lack health insurance.

^c^ Indicates the number of primary care physicians per 100 000 population.

### County-Level Age-Adjusted Screening Rates and SVI

**Figure 1.**
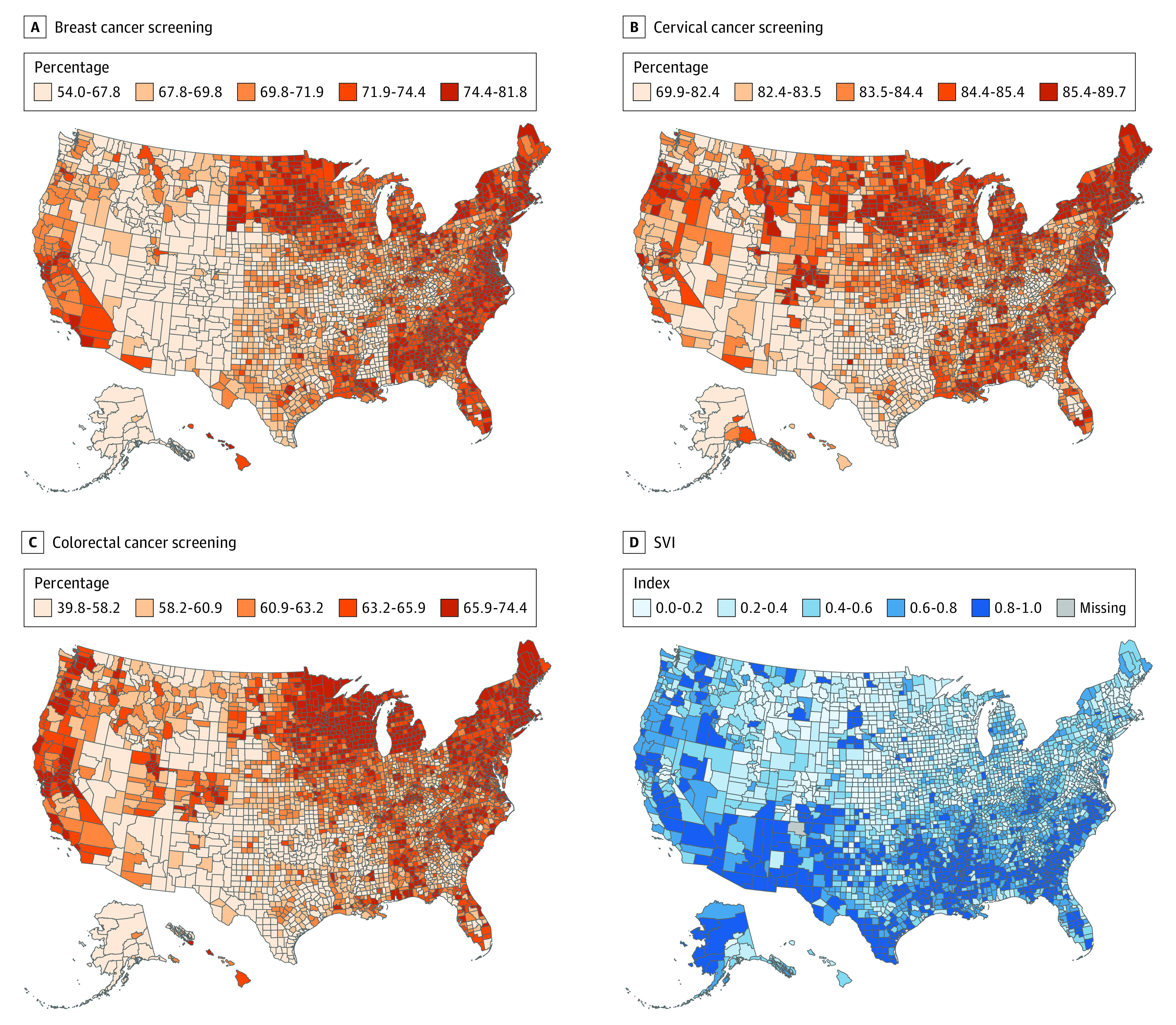
County-Level Breast, Cervical, and Colorectal Cancer Screening Rates and Social Vulnerability Index (SVI) in 2018 Maps are colored based on quintile cutoffs.

The mean (SD) county-level breast cancer screening rate was 70.8% (4.0%) and ranged from 54.0% (McKinley County, New Mexico) to 81.8% (Jefferson County, Alabama). The mean (SD) county-level cervical cancer screening rate was 83.9% (1.9%) and ranged from 69.9% (Kalawao County, Hawaii) to 89.7% (Cumberland County, Maine). The mean (SD) county-level colorectal cancer screening rate was 61.9% (4.8%) and ranged from 39.8% (Kusilvak County, Alaska) to 74.4% (Newport County, Rhode Island). Cancer screening rates presented substantial geographic variations, with all 3 screening rates being higher among the counties on the east and west coasts and lower in the South and Midwest. Maps of the screening rates and SVI categories presented clear geographic patterns, as counties with a higher SVI score also tended to have lower screening rates (Figure 1).

### Multivariable Bayesian Mixed-Effects Beta Model

**Figure 2.**
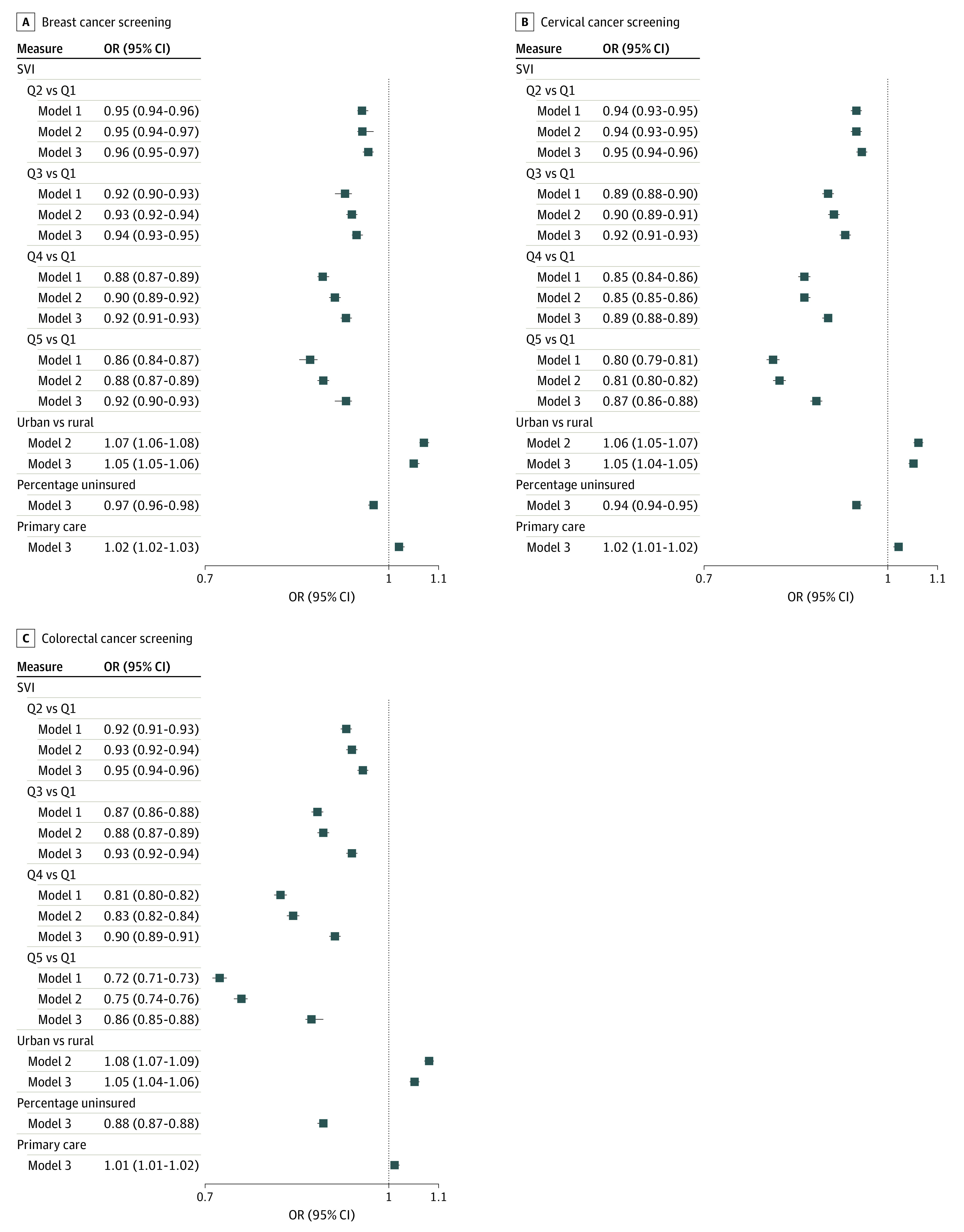
Odds Ratios (ORs) and Adjusted ORs for Breast, Cervical, and Colorectal Cancer Screening in 2018 Odds ratios were estimated by fitting a bayesian mixed-effects beta regression model. Percent uninsured and primary care rates were coded as continuous variables and scaled so ORs are associated with a 1-SD increase. Percentage uninsured indicates the proportion of adults lacking health insurance; primary care, the number of primary care physicians per 100 000 population. Model 1 included SVI-Q1 to Q5 and adjusted for the eligible population (percentage of population for the given outcome in the county). Model 2 included model 1 variables and further adjusted for urban-rural status; model 3 included model 2 variables and further adjusted for uninsured population and primary care.

Figure 2 presents the results from the bayesian beta regression models. With model 1 only adjusting for the eligible population size, counties with higher SVI showed a significant negative association with cancer screening rates. The odds of having cancer screening in the most vulnerable group (SVI-Q5) compared with the least vulnerable group (SVI-Q1) was 14% lower for breast cancer (OR, 0.86; 95% CrI, 0.84-0.87), 20% lower for cervical cancer screening (OR, 0.80; 0.79-0.81), and 28% lower for colorectal cancer screening (OR, 0.72; 0.71-0.73). In model 2, additionally adjusting for the county urban-rural status, the odds of having cancer screening was still significantly lower in SVI-Q5 than SVI-Q1 for all breast cancer (adjusted OR [aOR], 0.88; 95% CrI, 0.87-0.89), cervical cancer (aOR, 0.81; 95% CrI, 0.80-0.82), and colorectal cancer (aOR, 0.75; 95% CrI, 0.74-0.76). In the fully adjusted model (model 3), higher SVI groups continued to have significantly lower odds of screening than SVI-Q1 in all types of cancer screening, with the lowest odds in SVI-Q5. SVI-Q5 counties had 8% lower odds for screening compared with the SVI-Q1 counties (aOR, 0.92; 95% CrI, 0.90-0.93) in breast cancer screening. The aOR comparing SVI-Q5 with SVI-Q1 in cervical cancer screening was 0.87 (95% CrI, 0.86-0.88). In colorectal cancer screening, counties with the highest SVI were found to have 14% lower odds of screening than SVI-Q1 counties (aOR, 0.86; 95% CrI, 0.85-0.88). With SVI increased from Q2 to Q5, the decline appeared steeper for colorectal cancer screening compared with other cancer screenings.

Estimated ORs of screening rates associated with SVI did not show large changes in model 1, 2, or 3. However, for SVI-Q3 through SVI-Q5 compared with SVI-Q1, ORs were slightly attenuated in model 3 when additionally adjusting for urban-rural status, percentage of the population that was uninsured, and access to primary care. When comparing SVI-Q2 with SVI-Q1, sequentially adjusted models did not show any statistically significant changes. The complete results of all models can be found in the eTable in the Supplement.

Additional analyses between each individual SDoH used in SVI construction and each cancer screening uptake showed that, depending on how each individual SDoH was included in the analysis, the estimated association was substantially different. This finding illustrates the complex nature of SDoHs and cancer screening disparities and further corroborates the use of a composite score such as SVI (see eResults and eFigures 2-7 in the Supplement for details).

## Discussion

Using the PLACES data set, we found that the current USPSTF-recommended cancer screening rates for breast, cervical, and colorectal cancers were still suboptimal and presented substantial geographic disparity. This cross-sectional study also identified a significant association between area-level, multidimensional social vulnerability score and cancer screening uptake. Even after adjusting for county-level access to care, urban-rural status, and percentage of the population that was uninsured, the association remained consistent.

Previous studies investigated the association between cancer screening rates and area deprivation index in several US regions.^38,39^ Area deprivation index comprises area-level socioeconomic status (income, poverty, education, and employment), housing, transportation, and household composition.^40^ These studies found that a higher area deprivation index was associated with lower breast, cervical, or colorectal cancer screening rates. In the current study using SVI, which additionally includes area-level factors on racial and ethnic minority groups, language barriers, disability, and more, we were able to incorporate more diverse aspects to measure structural disparities associated with racial, ethnic, linguistic, and ability diversity. We note that these additional components are undeniably important to consider in developing interventions and policies for improving population health and reducing health disparities in the US.

The cancer screening disparities associated with racial, ethnic, linguistic, and ability diversity are well reported in previous studies.^18,19,20,22,23,24,26,27,28,30,41,42,43,44^ These factors were significantly associated with cancer screening rates when measured at an area level as well as individual level. Ecological associations between the area-level SDoH and cancer screening rates have been shown to be significant, and this association was consistent over different types of preventive health services.^17,45,46^ While contextual SDoH representing an individual domain of the social-ecological framework (eg, poverty or lack of education) had been studied previously with an established association to cancer screening disparity, a composite measure of social vulnerability provided a comprehensive look at differences in county-level cancer screening rates. Assessing area-level social vulnerability encompassing various SDoH allows us to capture conditions that affect all individuals living in the same area, either from compositional or contextual effects.^47,48^

Ecological analysis of geographic variation is instrumental in monitoring health disparities to inform and design area-based interventions to improve health equity. The area-based measures are intuitive for understanding health disparities and easy to use for identifying and locating targeted interventions for underserved populations.^49,50^ A composite index, like SVI, constructed by multiple SDoH closely associated with health care use and health outcomes provides a more holistic approach to understanding and estimating cancer screening uptake patterns than assessing individual determinants separately. Using SVI can be beneficial in implementing regionally targeted interventions for reducing disparities and improving overall cancer screening uptake. For example, interventions could be differently designed, and resources could be differently distributed based on cancer screening rates and SVI scores (eg, programs could be different in counties with low screening rates and low SVI than in counties with low screening rates and high SVI). Further research is warranted to evaluate the use of area-level SVI for developing and incorporating tailored interventions.

The Healthy People 2030 objectives set the target screening rates at 77.1%, 84.3%, and 74.4% for breast, cervical, and colorectal cancer, respectively.^7,8,9^ Of the 3141 counties in this study, 2992 (95.2%) are currently under target rates for breast cancer screening and 1787 (56.9%) for cervical cancer screening. All but 1 county out of 3141 has met the colorectal cancer screening rate. Maps of those counties that have not yet achieved the Healthy People 2030 cancer screening target rates are presented in eFigure 8 in the Supplement. The county-level average cervical cancer screening rate (84%) was comparatively higher than the colorectal cancer screening (62%) or breast cancer screening (71%) rates (eFigure 9 in the Supplement). Considering colorectal and cervical cancer screenings are both graded A, the substantially lower colorectal cancer screening rate is worth attention. Previous studies identified the invasiveness of the procedure (eg, endoscopy) and its associated fear as notable barriers to colorectal screening tests, which might explain the low rates.^51,52^ Given the different modalities of colorectal screening (endoscopy vs fecal occult blood test) and their accessibility or invasiveness, more studies are needed to identify the most effective interventions for improving colorectal cancer screening uptake and reducing disparities across different degrees of vulnerability. Cervical cancer screening uptake is the highest among the 3 screenings on average, yet with substantial disparity across counties; reducing this disparity might be the priority of many population health researchers, along with improving overall uptake. In fact, a recent study reported significantly higher cervical cancer incidence rates among low-income counties.^53^ Moreover, the previous decline in cervical cancer incidence has reversed and started to increase among these low-income counties,^53^ suggesting a possible impact of area-level cervical cancer screening disparities by socioeconomic status. It is also known that cervical cancer screening rates have been declining in recent years among eligible women, including those who could not benefit from the human papillomavirus vaccine.^54^ Further research on assessing the optimal interventions to reduce disparities and improve uptake in cervical cancer screening is crucial to reverse this increasing incidence trend in less affluent areas and overall declining screening rates.

Moreover, the COVID-19 pandemic was associated with an overall large cancer screening deficit in the US.^55^ Studies have also indicated that socially vulnerable populations were disproportionately affected by the pandemic.^56,57^ Further research investigating differential effects of SVI on the association between the pandemic and cancer screening rates would be beneficial for extensively understanding how social vulnerability can impact the health of communities.

### Strengths and Limitations

This study has several strengths. First, using the newly released PLACES project, we were able to examine the geospatial pattern of 3 cancer screening rates in all US counties. Second, we performed extensive analyses on each area-level SDoH and composite SVIs and their associations with cancer screening uptake. By doing so, we provided the rationale and demonstrated an analytical procedure for performing an ecological study to investigate the contextual factors and health outcomes.

It would be remiss of us to ignore the limitations of our analysis. First, the cancer screening rates used in this analysis were based on the Behavioral Risk Factor Surveillance System, which was self-reported and hence subject to bias in self-reporting. However, the Behavioral Risk Factor Surveillance System has been used as the key data source for informing Healthy People screening plans and for the CDC’s periodical reports.^58^ Second, we conducted an ecological study, which cannot be used to assess individual-level screening behavior and social vulnerability. Third, we acknowledge that the county-level analysis might mask more granular issues within a county. Owing to the lack of age-adjusted cancer screening rate data at a more granular level (ie, Census tract level) in the current PLACES data, we only conducted the analysis at the county level. Future research examining smaller area units is needed to better understand the relationships between social vulnerability and cancer screening. Despite these limitations, our study adds to the literature by providing key information for cancer surveillance and identification of geographic areas that present substantial disparities in cancer screening.

## Conclusions

This cross-sectional study found that US counties with higher social vulnerability had lower rates of USPSTF-recommended cancer screenings. These findings add to a growing body of evidence on the influence of area-level SDoH context on population cancer prevention efforts. Geographically targeted public health interventions could be further informed and improved by a composite measure reflecting the multidimensional nature of SDoH. More efforts are still needed to better incorporate the SVI tool into assessing geographic disparities in cancer outcomes.

## Notes

Supplement. **eFigure 1.** Maps of the US county-level Rural-Urban Continuum Codes (RUCC), percentage of uninsured population and access to primary physicians **eMethods.** Model equations and details **eTable.** Association of SVI and three cancer screening rates using 2018 PLACES data **eResults.** Additional analysis for PLACES cancer screening rates and SVI **eFigure 2.** Association between individual SDoH and cervical cancer screening rates (%) **eFigure 3.** Association between individual SDoH and breast cancer screening rates (%) **eFigure 4.** Association between individual SDoH and colorectal cancer screening rates (%) **eFigure 5.** Association between SDoH and cervical cancer screening rates (%) with all SDoH in the model **eFigure 6.** Association between SDoH and breast cancer screening rates (%) with all SDoH in the model **eFigure 7.** Association between SDoH and colorectal cancer screening rates (%) with all SDoH in the model **eFigure 8.** Maps identifying US counties that are currently not meeting the Healthy People 2030 target for the three cancer screening rates **eFigure 9.** US County-level maps of three cancer screening rates using national average as cutoff points
