# Supplementary material for: County-Level Social Vulnerability and Breast, Cervical, and Colorectal Cancer Screening Rates in the US, 2018
Source: JAMA Netw Open. 2022 Sep 27;5(9):e2233429. doi: 10.1001/jamanetworkopen.2022.33429 (PMC9516325; doi:10.1001/jamanetworkopen.2022.33429)
Supplement: Supplement. — eFigure 1. Maps of the US county-level Rural-Urban Continuum Codes (RUCC), percentage of uninsured population and access to primary physicians eMethods. Model equations and details eTable. Association of SVI and three cancer screening rates using 2018 PLACES data eResults. Additional analysis for PLACES cancer screening rates and SVI eFigure 2. Association between individual SDoH and cervical cancer screening rates (%) eFigure 3. Association between individual SDoH and breast cancer screening rates (%) eFigure 4. Association between individual SDoH and colorectal cancer screening rates (%) eFigure 5. Association between SDoH and cervical cancer screening rates (%) with all SDoH in the model eFigure 6. Association between SDoH and breast cancer screening rates (%) with all SDoH in the model eFigure 7. Association between SDoH and colorectal cancer screening rates (%) with all SDoH in the model eFigure 8. Maps identifying US counties that are currently not meeting the Healthy People 2030 target for the three cancer screening rates eFigure 9. US County-level maps of three cancer screening rates using national average as cutoff points [file jamanetwopen-e2233429-s001.pdf]

Supplemental Online Content

Bauer C, Zhang K, Xiao Q, Lu J, Hong YR, Suk R. County-level social vulnerability and breast, cervical, and colorectal cancer screening rates in the US, 2018. *JAMA Netw Open*. 2022;5(9):e2233429. doi:10.1001/jamanetworkopen.2022.33429

eFigure 1. Maps of the US county-level Rural-Urban Continuum Codes (RUCC), percentage of uninsured population and access to primary physicians
eMethods. Model equations and details
eTable. Association of SVI and three cancer screening rates using 2018 PLACES data
eResults. Additional analysis for PLACES cancer screening rates and SVI
eFigure 2. Association between individual SDoH and cervical cancer screening rates (%)
eFigure 3. Association between individual SDoH and breast cancer screening rates (%)

eFigure 4. Association between individual SDoH and colorectal cancer screening rates (%)
eFigure 5. Association between SDoH and cervical cancer screening rates (%) with all SDoH in the model
eFigure 6. Association between SDoH and breast cancer screening rates (%) with all SDoH in the model
eFigure 7. Association between SDoH and colorectal cancer screening rates (%) with all SDoH in the model
eFigure 8. Maps identifying US counties that are currently not meeting 2023 Healthy People target for the three cancer screening rates
eFigure 9. US County-level maps of three cancer screening rates using national average as cutoff points

This supplemental material has been provided by the authors to give readers additional information about their work.

© 2022 Bauer C et al. *JAMA Network Open.*

eFigure 1. Maps of the US county-level Rural-Urban Continuum Codes (RUCC), percentage of uninsured population and access to primary physicians (calculated as the # of primary physicians per 100,000 population).

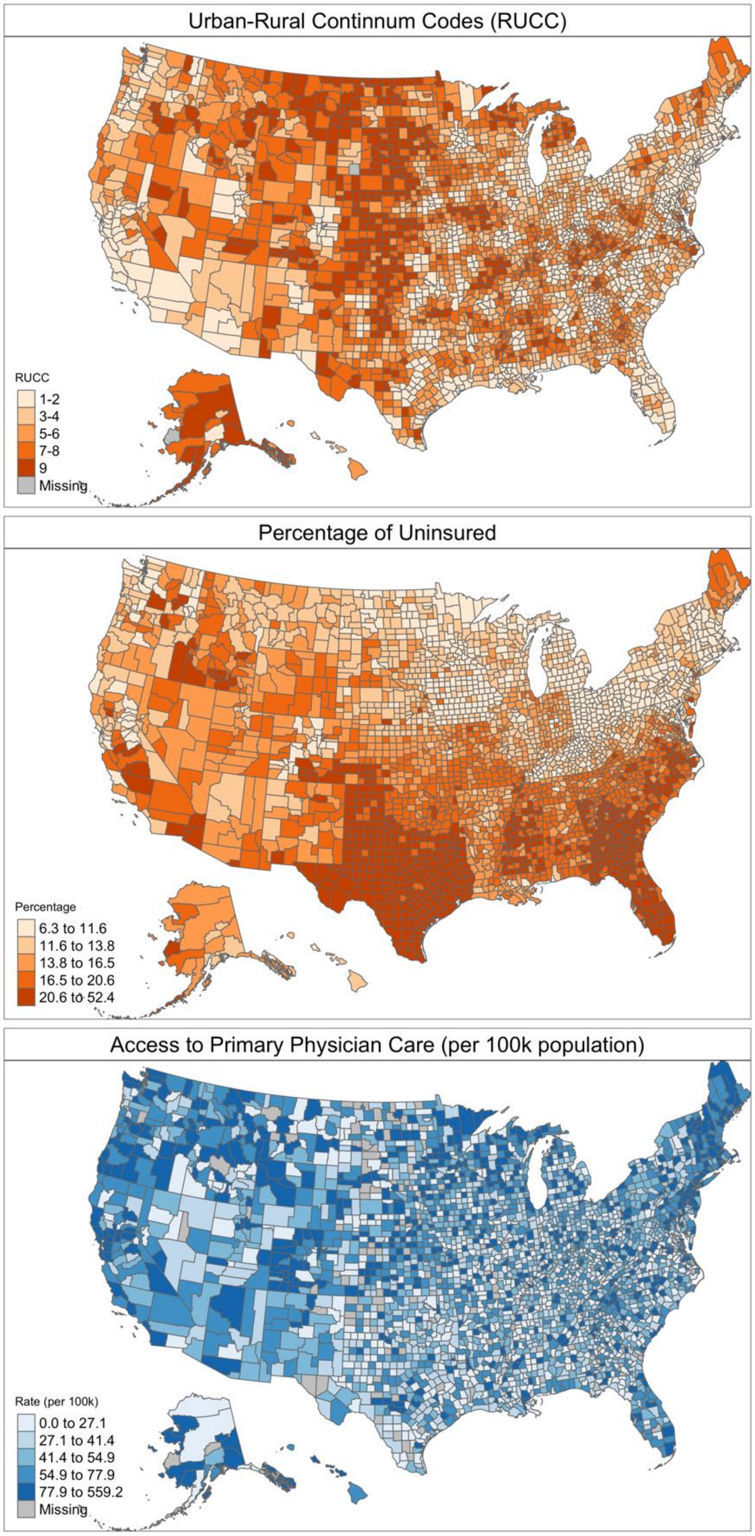

© 2022 Bauer C et al. *JAMA Network Open.*

eMethods. Model equations and details.

We used a Bayesian mixed-effect Beta model to evaluate the association between county-level cancer screening rates by type and SVI categories (i.e., Q1: 0-<0.2, Q2: 0.2-<0.4, Q3: 0.4-<0.6, Q4: 0.6-<0.8, Q5: 0.8-1). Because all three screening rates were reported in percentages bounded between 0 and 1, we chose a Beta distribution when modeling the rate p_i for the ith county. The model (Model 1) can be presented as:

$$\log\left(\frac{p_i}{1-p_i}\right) = \alpha + \boldsymbol{\beta_1}\text{State} + \boldsymbol{\beta_2}\text{SVI}_q + u_i + \epsilon_i, \qquad \epsilon_i \sim \text{Beta}(a, b)$$

We included a fixed effect $\boldsymbol{\beta_1}$ to account for the unmeasured state-level factors (e.g., policy difference), and a fixed effect $\boldsymbol{\beta_2}$ of the SVI categories denoted by SVI_q. Note that we used the bold font for these coefficient parameters to highlight that they were vectors - for example, there were 4 parameters in $\boldsymbol{\beta_2}$, with one each from SVI Q2, Q3, Q4 and Q5, where SVI Q1 was used as the reference category. We included a county-level random effect u_i to account for the effect from any additional unmeasured county-level factor on the cancer screening rates, and assumed a normal distribution for it. The residuals were assumed to have a beta distribution with parameters a and b. Our primary interest was the coefficient $\boldsymbol{\beta_2}$, which was presented as the odds ratio relative to the reference group Q1. We used the 95% posterior credible intervals (95% CrI) to assess the statistical significance of SVI_q.

We considered the following model as Model 2:

$$\log\left(\frac{p_i}{1-p_i}\right) = \alpha + \boldsymbol{\beta_1}\text{State} + \boldsymbol{\beta_2}\text{SVI}_q + \beta_3\text{Urban} + u_i + \epsilon_i,$$

where we further included the urban/rural indicator variable as the adjustment variables.

In Model 3, we additionally adjusted for the percentage of uninsured (Uninsured) and access to primary care (PrimaryCare), both were scaled to have mean 0 and SD 1.

$$\log\left(\frac{p_i}{1-p_i}\right) = \alpha + \boldsymbol{\beta_1}\text{State} + \boldsymbol{\beta_2}\text{SVI}_q + \beta_3\text{Urban} + \beta_4\text{Uninsured} + \beta_5\text{PrimaryCare} + u_i + \epsilon_i.$$

We presented the results from all three models.

© 2022 Bauer C et al. *JAMA Network Open.*

eTable. Association of SVI and three cancer screening rates using 2018 PLACES data. Odds ratio (OR) and the 95% posterior credible intervals (95% CrIs) were obtained using Bayesian mixed-effect Beta regression models.

	Breast cancer screening			Cervical cancer screening			Colorectal cancer screening		
	Model 1	Model 2	Model 3	Model 1	Model 2	Model 3	Model 1	Model 2	Model 3
(Intercept)	2.22 (2.13, 2.32)	2.06 (1.98, 2.15)	1.97 (1.89, 2.05)	3.82 (3.63, 4.02)	4.16 (3.95, 4.38)	4.31 (4.11, 4.52)	1.65 (1.58, 1.73)	1.48 (1.41, 1.55)	1.34 (1.29, 1.4)
SVI Q1	Reference	Reference	Reference	Reference	Reference	Reference	Reference	Reference	Reference
SVI Q2	0.95 (0.94, 0.96)	0.95 (0.94, 0.97)	0.96 (0.95, 0.97)	0.94 (0.93, 0.95)	0.94 (0.93, 0.95)	0.95 (0.94, 0.96)	0.92 (0.91, 0.93)	0.93 (0.92, 0.94)	0.95 (0.94, 0.96)
SVI Q3	0.92 (0.90, 0.93)	0.93 (0.92, 0.94)	0.94 (0.93, 0.95)	0.89 (0.88, 0.90)	0.90 (0.89, 0.91)	0.92 (0.91, 0.93)	0.87 (0.86, 0.88)	0.88 (0.87, 0.89)	0.93 (0.92, 0.94)
SVI Q4	0.88 (0.87, 0.89)	0.9 (0.89, 0.91)	0.92 (0.91, 0.93)	0.85 (0.84, 0.86)	0.85 (0.85, 0.86)	0.89 (0.88, 0.89)	0.81 (0.80, 0.82)	0.83 (0.82, 0.84)	0.90 (0.89, 0.91)
SVI Q5	0.86 (0.84, 0.87)	0.88 (0.87, 0.89)	0.92 (0.90, 0.93)	0.80 (0.79, 0.81)	0.81 (0.80, 0.82)	0.87 (0.86, 0.88)	0.72 (0.71, 0.73)	0.75 (0.74, 0.76)	0.86 (0.85, 0.88)
Urban		1.07 (1.06, 1.08)	1.05 (1.05, 1.06)		1.06 (1.05, 1.07)	1.05 (1.04, 1.05)		1.08 (1.07, 1.09)	1.05 (1.04, 1.06)
%Uninsured			0.97 (0.96, 0.97)			0.94 (0.94, 0.95)			0.88 (0.87, 0.88)
Primary Care			1.02 (1.02, 1.03)			1.02 (1.01, 1.02)			1.01 (1.01, 1.02)

Urban: Urban variable is a binary indicator for county level urban-rural status. It is derived from U.S. Department of Agricultural Rural-Urban Continuum Codes.
Uninsured %: Proportion of current lack of health insurance among adults aged 18-64 years.
Primary Care: Number of primary care physicians per 100,000 population.
Model 1: included SVI Q1-Q5 and adjusted for the eligible population (percentage of population for the given screening outcome in the county)
Model 2: included variables in Model 1 and further adjusted for urban/rural status
Model 3: include all variables in Model 2 and further adjusted for % of uninsured population and measures of access to primary care physicians.
Abbreviations: SVI, social vulnerability index; OR, odds ratio; CrI, Bayesian credible intervals.

© 2022 Bauer C et al. *JAMA Network Open.*

eResults. Additional Analysis For PLACES Cancer Screening Rates and Social Vulnerability Index

Many contextual social determinants of health (SDoH) have been demonstrated as risk factors to many health related outcomes and behaviors. Individuals SDoHs, such as % of population living under poverty and % of population unemployed, have been extensively studied and established as having adverse effect to population health.

This analysis used county-level 15 SDoH variables from 3,142 counties, and quantified their associations with three cancer screening rates (cervical cancer screening, breast cancer screening, and colorectal cancer screening). The cancer screening rates were extracted from 2018 PLACES project, which were model-based estimates using BRFSS database. The 15 SDoH variables selected as they were used in the construction of social vulnerabilities index (SVI) by CDC. The purpose of this analysis to investigate the association between *individual* SDoH and the three cancer screening rates at the US county level.

For each cancer screening outcome, we divided the particular SDoH under study to quintiles Q1 – Q5, with Q1 as the lowest quintile and the reference group in the analysis. We used a linear regression model, where we adjusted the eligible population size given the relevancy of the type of cancer screening, and included the State as a fixed effect.

Supplementary Figures 7-9 present the estimated association between each of the cancer screening rates and individual SDoH. For each outcome, most of the individual SDoH presented statistically significant association with the cancer screening rates. Specifically, % of population living with crowded housing, disabled, living in poverty, as minority, with no high school diploma, with no vehicles, unemployed, and single parent house, and per capita income presented consistent and strong association with all three cancer screening outcomes.

Supplementary Figures 10-12 present the estimated association between each of the cancer screening rates, and the SDoH, when all SDoH were included simultaneously in the model. It was clear that effects of the SDoH now were highly attenuated, and some no longer showed any statistical significance. More importantly, some SDoH showed an opposite association direction compared to those in Supplementary Figures 5-7. For example, the % of minority showed a positive and statistically significant association with cervical cancer screening rate in Supplementary Figure 5, where counties with higher % of minority had decreased screening rates; however, this association was flipped in Supplementary Figure 8, where counties with higher % of minority had increased screening rates.

The reason for such opposite direction in estimated effects of the SDoH is speculated due to the high correlation between these contextual SDoH. Therefore, using a composite score, such as the SVI constructed using these SDoHs, may be better in capturing the difference of the county-level cancer screening rates due to the SDoHs.

© 2022 Bauer C et al. *JAMA Network Open.*

eFigure 2. Association between individual SDoH and cervical cancer screening rates (%). Each SDoH was divided into quintiles with Quintile 1 the lowest and used as the reference group. We fit a simple linear regression and extracted the coefficients. Black dots represent the point estimate from the regression model, and the bars represent the associated 95% confidence intervals.

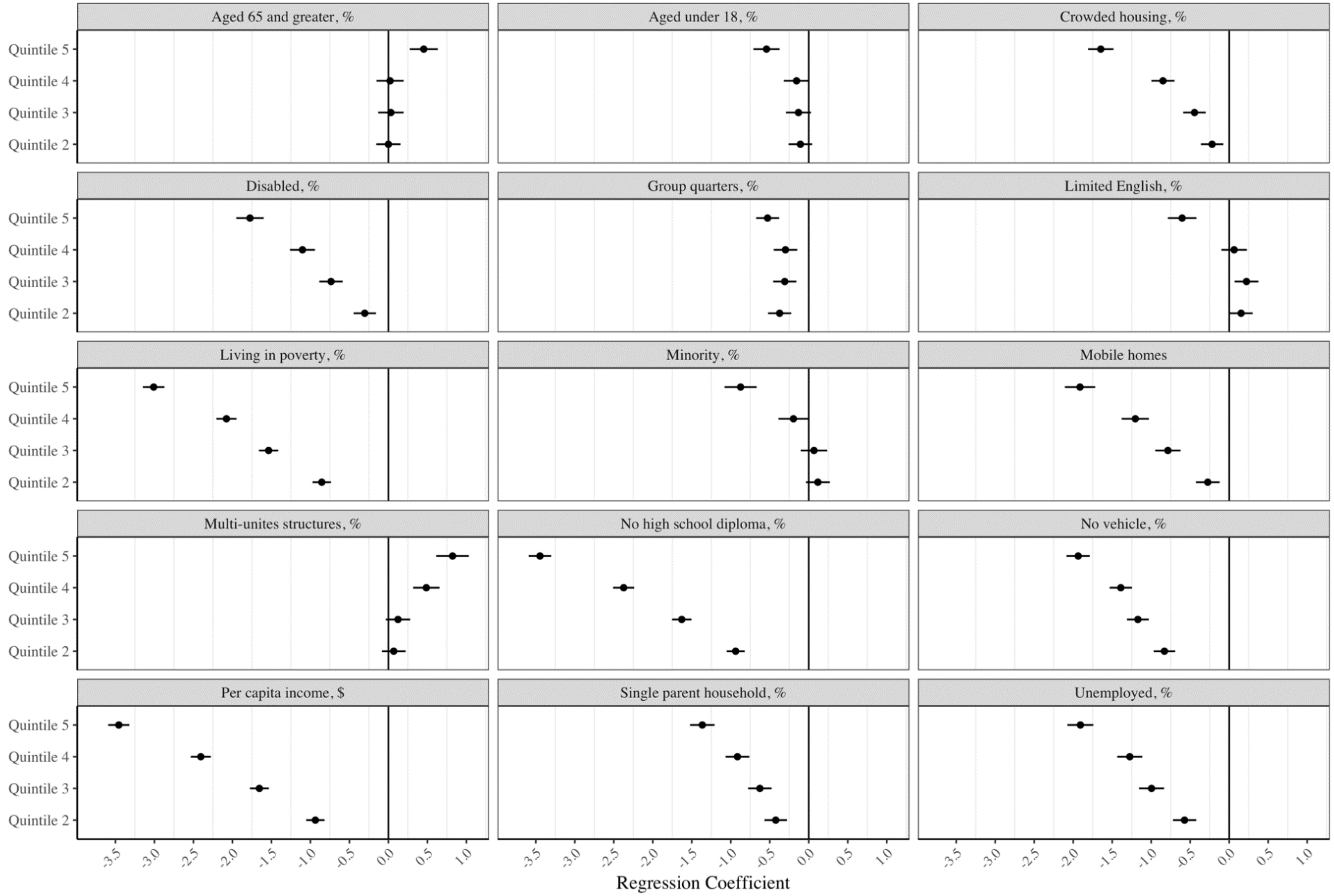

© 2022 Bauer C et al. *JAMA Network Open.*

eFigure 3. Association between individual SDoH and breast cancer screening rates (%). Each SDoH was divided into quintiles with Quintile 1 the lowest and used as the reference group. We fit a simple linear regression and extracted the coefficients. Black dots represent the point estimate from the regression model, and the bars represent the associated 95% confidence intervals.

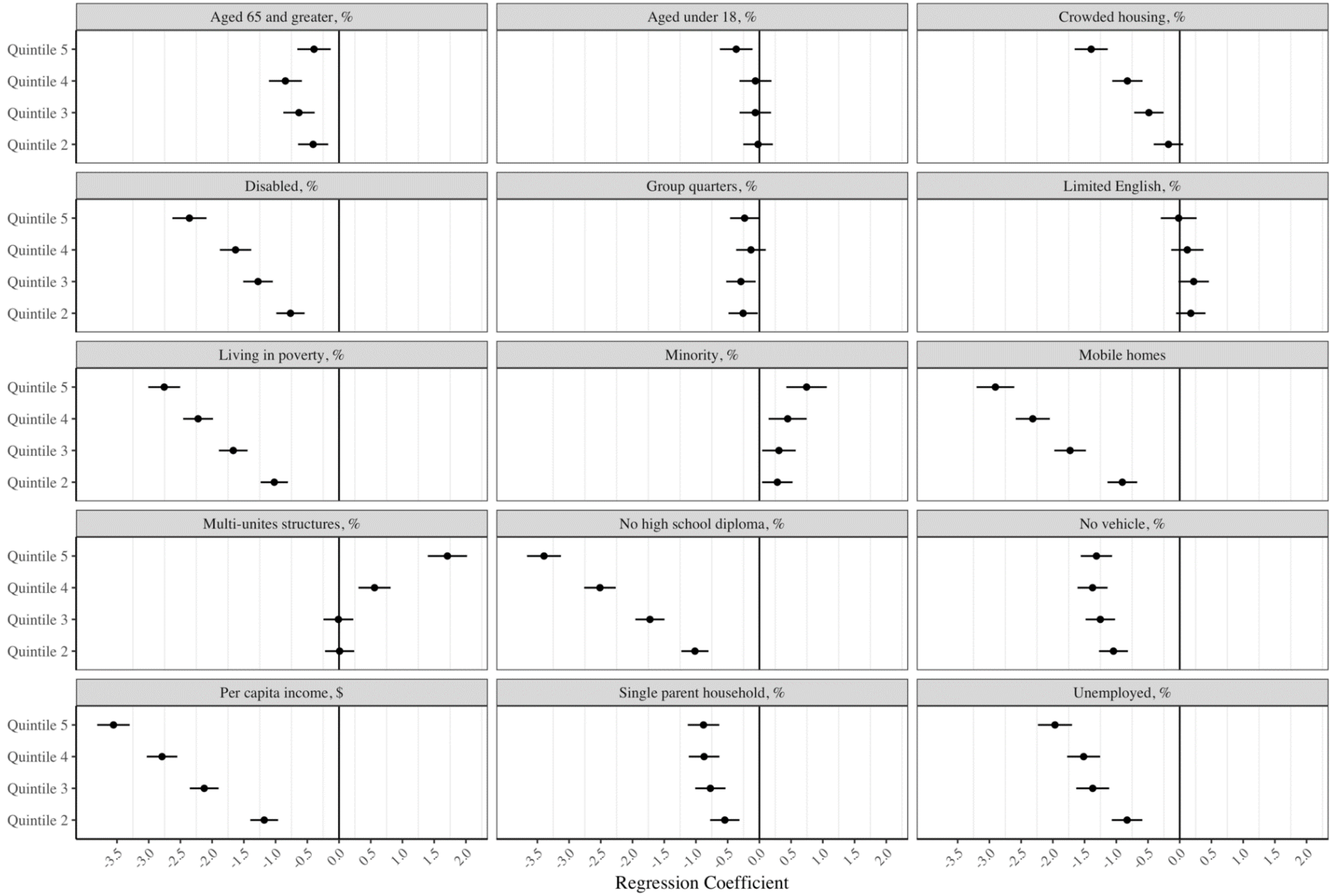

© 2022 Bauer C et al. *JAMA Network Open.*

eFigure 4. Association between individual SDoH and colorectal cancer screening rates (%). Each SDoH was divided into quintiles with Quintile 1 the lowest and used as the reference group. We fit a simple linear regression and extracted the coefficients. Black dots represent the point estimate from the regression model, and the bars represent the associated 95% confidence intervals.

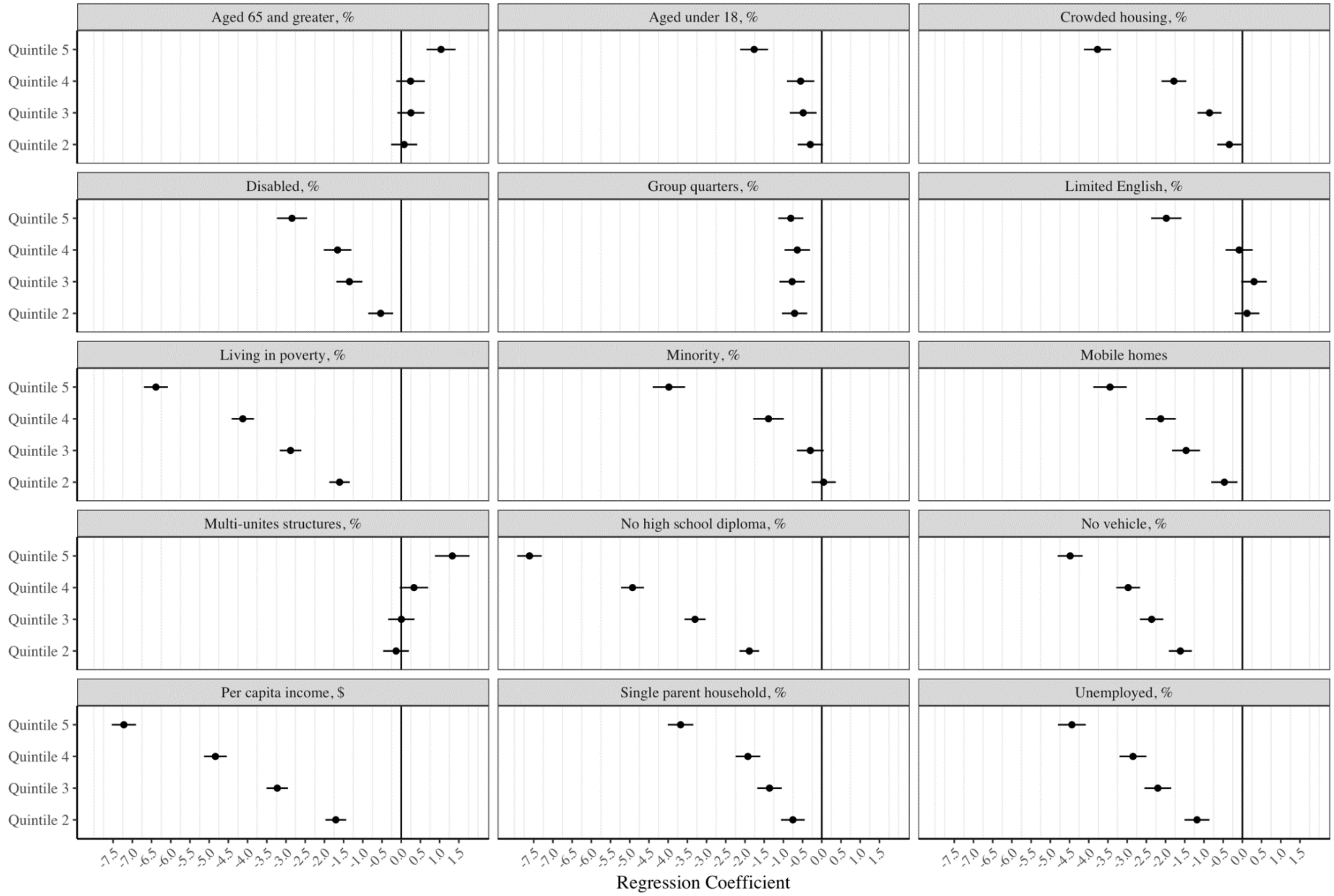

© 2022 Bauer C et al. *JAMA Network Open.*

eFigure 5. Association between SDoH and cervical cancer screening rates (%). All 15 SDoH variables were simultaneously included in the model. Each SDoH was divided into quintiles with Quintile 1 the lowest and used as the reference group.

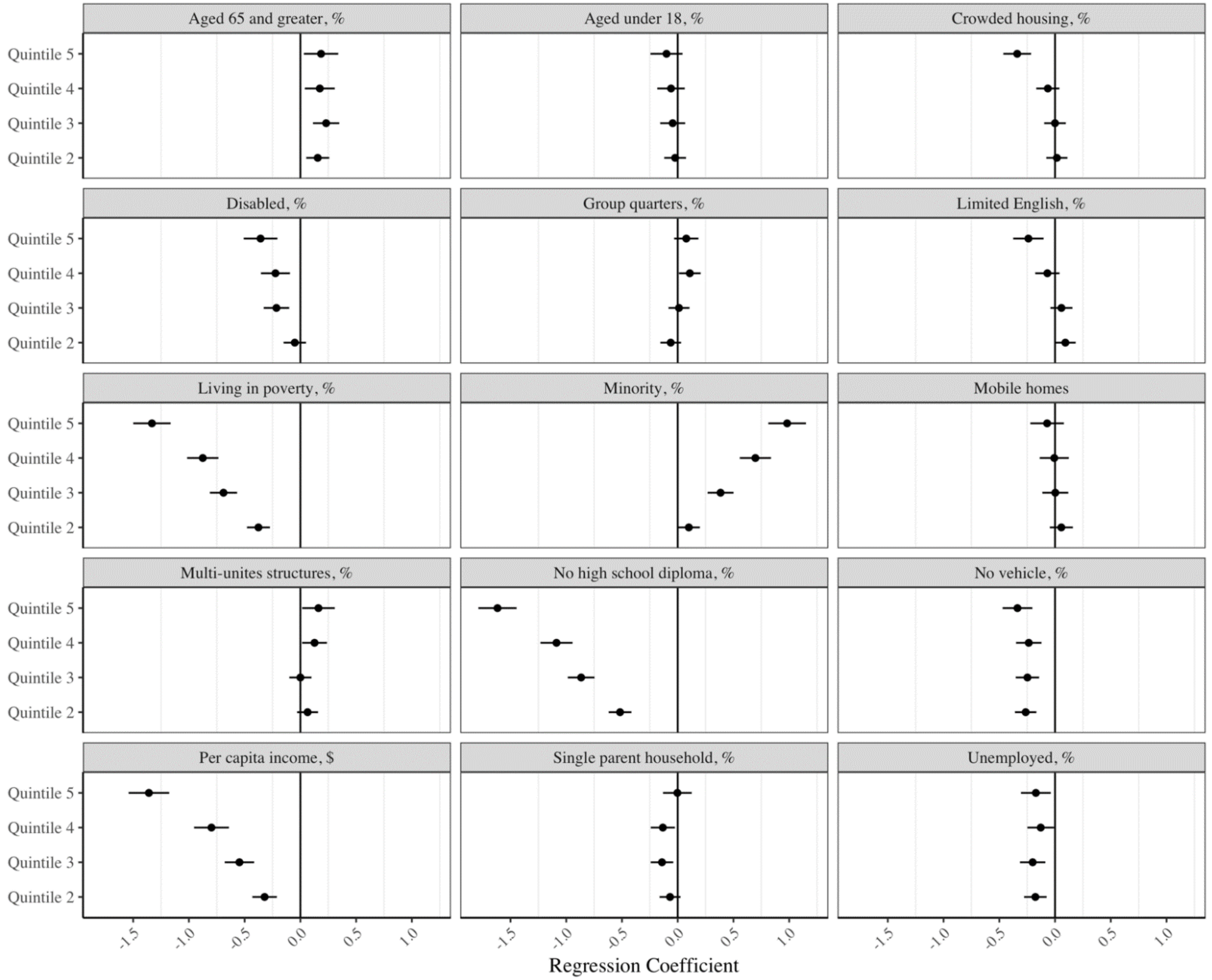

© 2022 Bauer C et al. *JAMA Network Open.*

eFigure 6. Association between SDoH and breast cancer screening rates (%). All 15 SDoH variables were simultaneously included in the model. Each SDoH was divided into quintiles with Quintile 1 the lowest and used as the reference group.

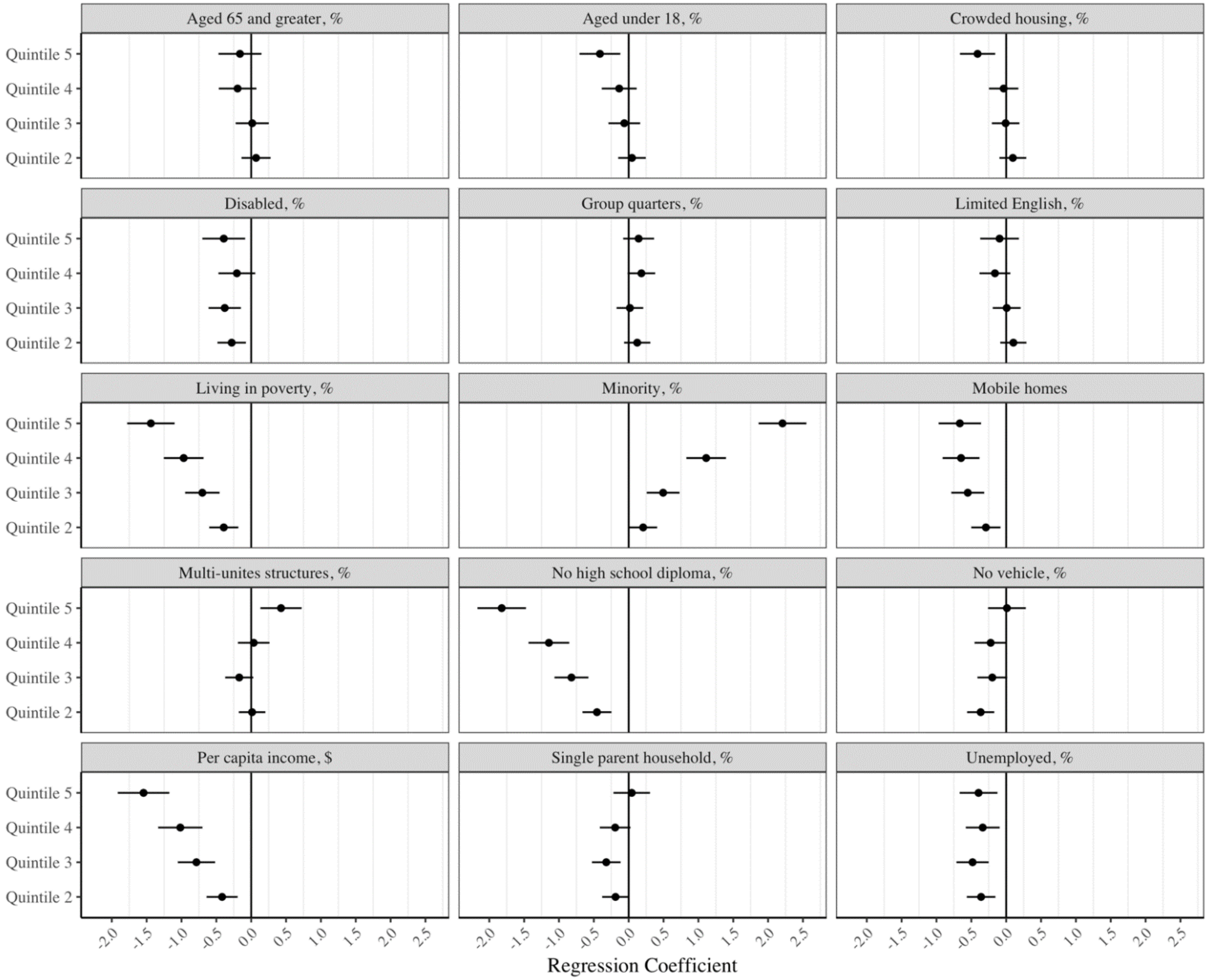

© 2022 Bauer C et al. *JAMA Network Open.*

eFigure 7. Association between SDoH and colorectal cancer screening rates (%). All 15 SDoH variables were simultaneously included in the model. Each SDoH was divided into quintiles with Quintile 1 the lowest and used as the reference group.

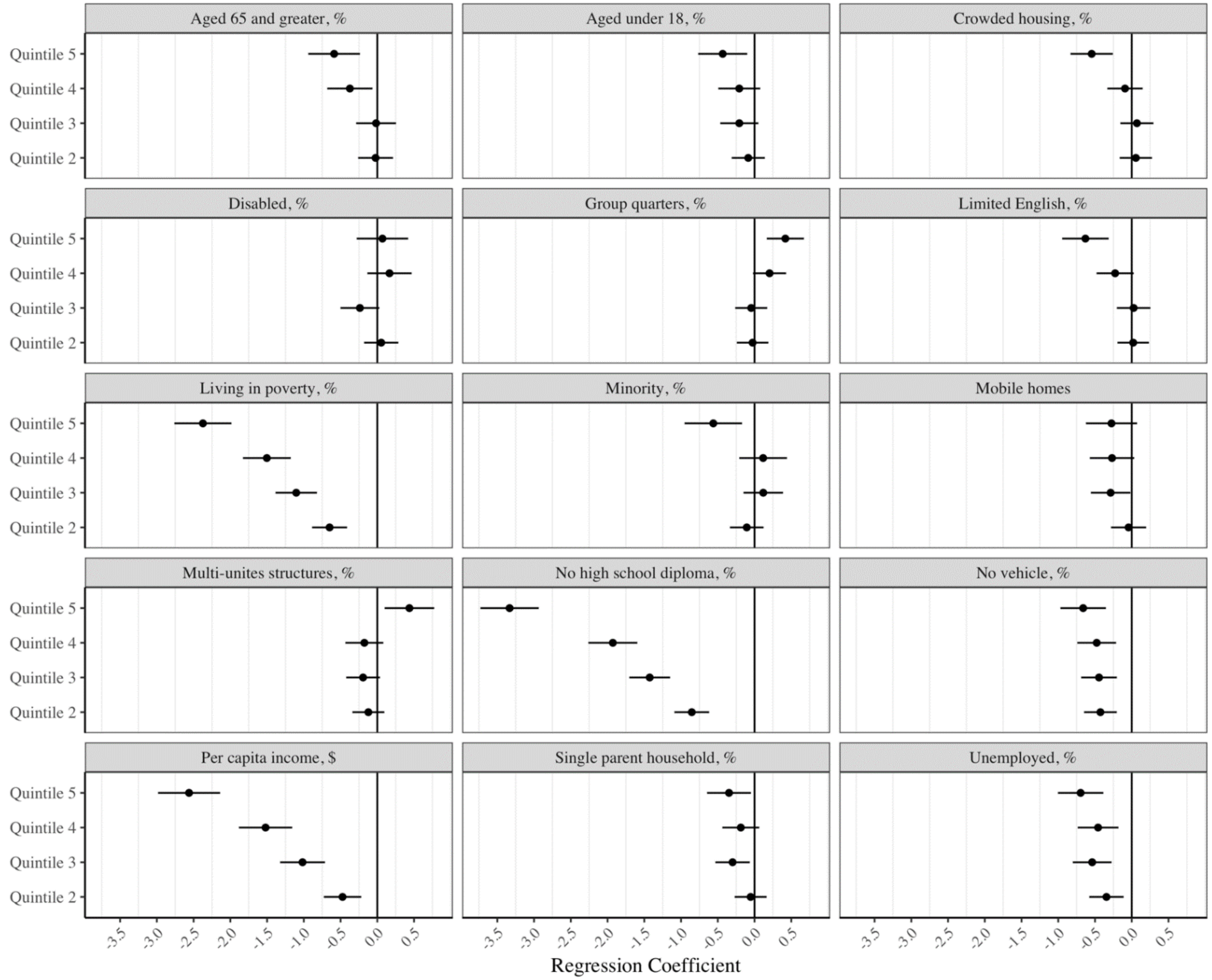

© 2022 Bauer C et al. *JAMA Network Open.*

eFigure 8. US County-level map of three cancer screening rates using 2023 Healthy People target as the cutoff point. Of the 3,142 counties, 2,992 (95.2%) are currently under Healthy People 2030 target rates for breast cancer screening and 1,787 (56.9%) for cervical cancer screening. All but one county (1 out of 3,142) has met Healthy People 2030 colorectal cancer screening targeted rate.

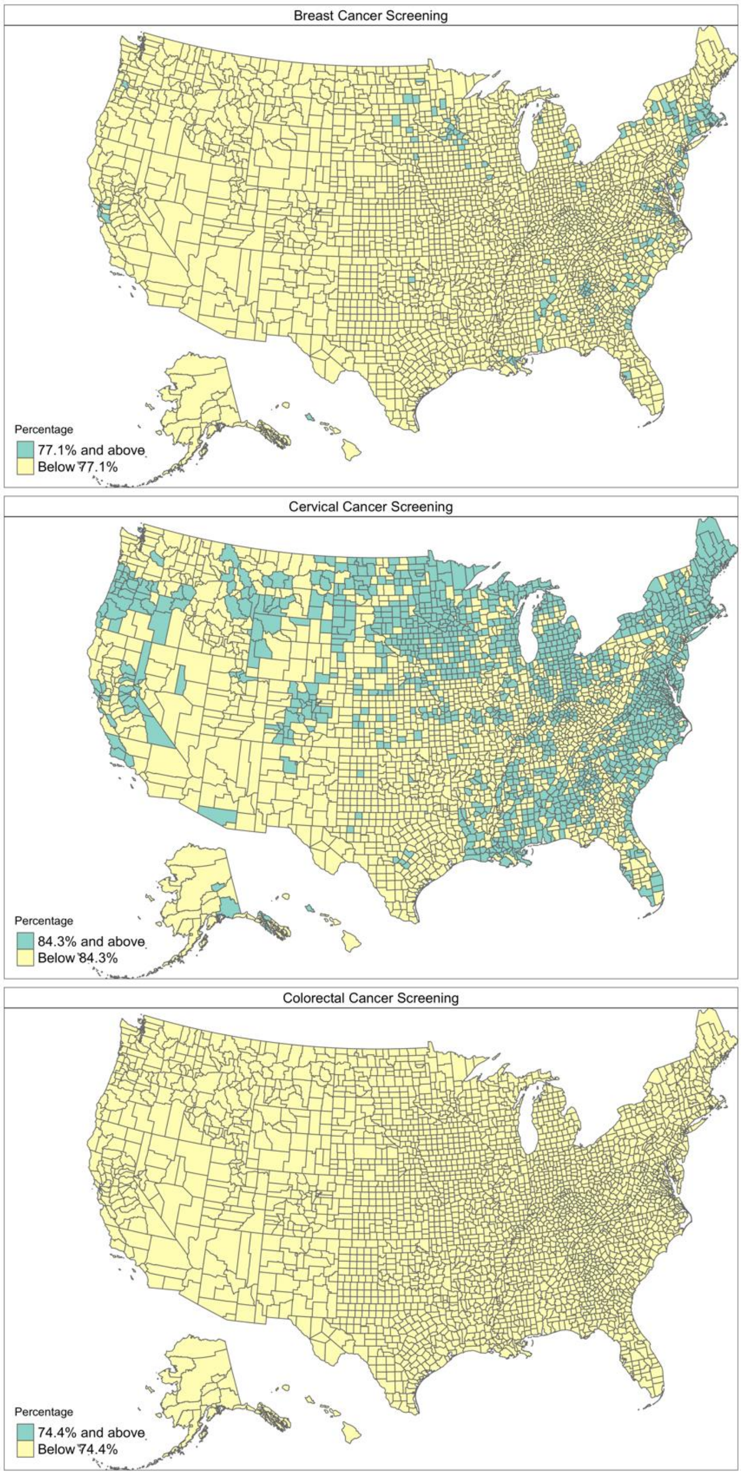

© 2022 Bauer C et al. *JAMA Network Open.*

eFigure 9. US County-level maps of three cancer screening rates using national average as cutoff points.

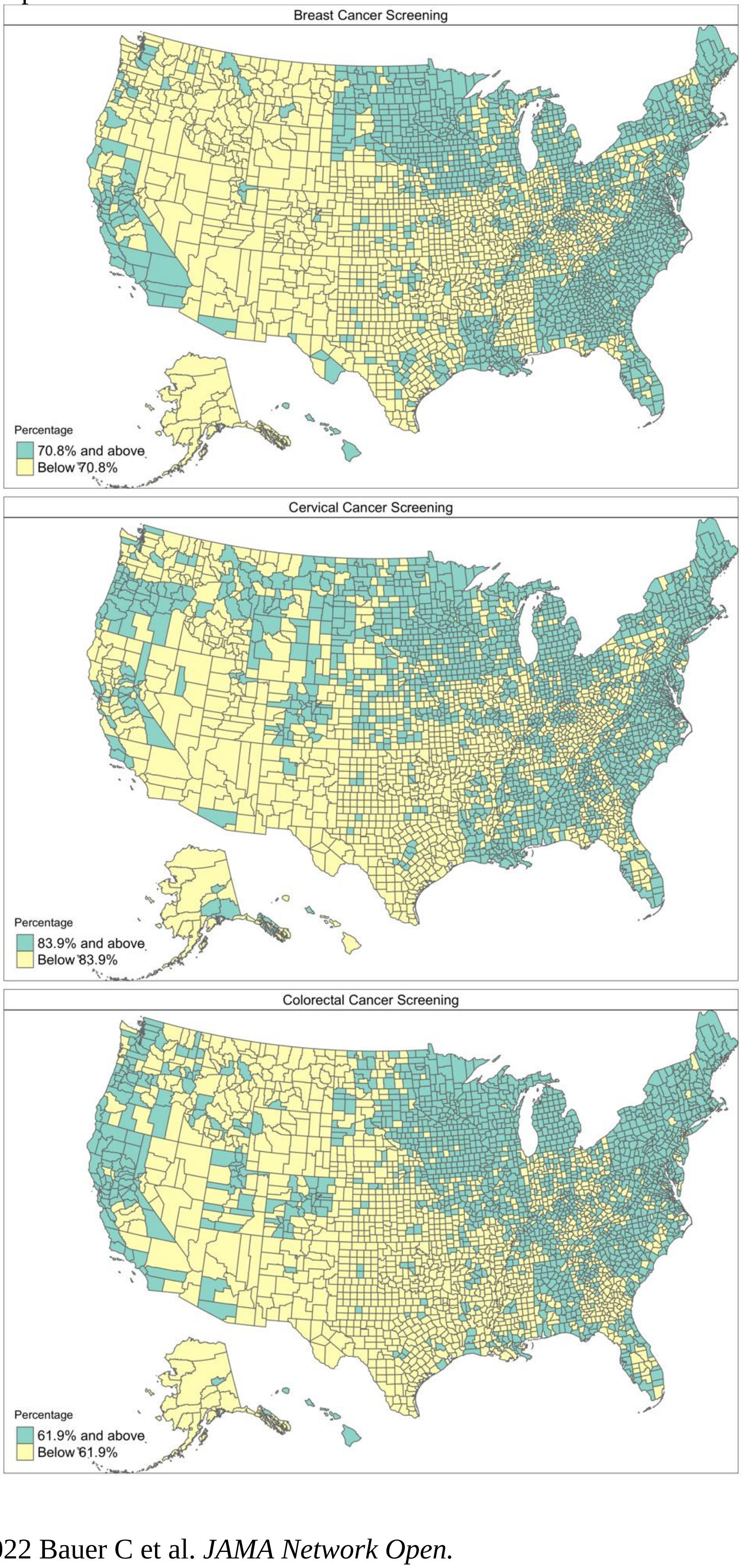

© 2022 Bauer C et al. *JAMA Network Open.*
